# Improving thermal conductivity of Al/SiC composites by post-oxidization of reaction-bonded silicon carbide preforms

**DOI:** 10.1038/s41598-024-67653-y

**Published:** 2024-07-18

**Authors:** Xinping Lin, Qiang Xu, Tianyou Deng, Bingquan Yang, Liang Chen

**Affiliations:** 1 Research and Development Center, BYD Company Limited, Dapeng District, Shenzhen, 518118 China; 2 New Materials Division, BYD Company Limited, Dapeng District, Shenzhen, 518118 China

**Keywords:** Thermodynamics, Ceramics, Composites, Metals and alloys

## Abstract

High thermal conductivity aluminium-based silicon carbide (Al/SiC) composites were successfully fabricated through post-oxidization of reaction-bonded silicon carbide preforms (RS preforms), utilizing vacuum pressure infiltration technology. The study investigated the regulation of interfacial reactions in conventional sintering and reaction sintering. Conventional sintering introduced a large amount of SiO_2_, which negatively impacted the thermal conductivity of the Al/SiC composite, but the reaction sintering not. The proposed post-oxidization treatment of RS preforms effectively removed residual carbon from the SiC particle surfaces, thereby forestalling the formation of Al_4_C_3_. Furthermore, the post-oxidization treatment effectively formed lightweight SiO_2_ deposits onto the surface of SiC particles, improving Al-SiC interfacial wettability and reducing thermal resistance, thereby enhancing composite thermal conductivity. Notably, the thermal conductivity of the post-oxidized sample exhibited an increase of 6.5% compared to the untreated sample. The study also evaluated the impact of particle size distribution on volume fraction and thermal properties. The optimized Al/SiC composites yielded thermal conductivity, coefficient of thermal expansion, bending strength, and Young’s modulus values of 237.3 W/m K, 8.5 × 10^−6^/°C, 325 MPa, and 75.9 GPa, respectively.

## Introduction

Metal matrix composites (MMCs) are engineered by integrating a continuous metallic matrix, typically metal alloys, with a discrete phase of reinforcement, such as ceramic particles, to attain customized material properties^1–4^. These materials have garnered significant attention, particularly for applications within the aerospace and automotive sectors, owing to their exceptional mechanical robustness and thermal characteristics^5,6^. Among MMCs, aluminium-based silicon carbide (Al/SiC) composites stand out^7–9^, with aluminium alloys serving as the matrix and SiC particles functioning as the reinforcing agent. Ongoing researches have revealed the versatility of Al/SiC composites, which are now employed across various industries, ranging from aerospace and biomedical to underwater technology, automotive, and electronics^10–13^. Their utility stems from their high thermal conductivity (TC), low coefficient of thermal expansion (CTE), superior corrosion resistance, coupled with ease of access and processability^10–13^.

The properties of Al/SiC composites are significantly influenced by several critical factors, including the interface microstructure, the size distribution and volume fraction of the reinforcing materials, as well as the fabrication methods^14,15^. The SiC preform produced via reaction sintering (RS) under oxygen-free conditions is commonly known as reaction-bonded silicon carbide preform (RS preform)^16,17^. This technology, introduced by Popper in the 1950s^16,18^, offers a potent means of controlling the interfacial chemistry in the production of Al/SiC composites. The crystal structures of SiC predominantly exist in two forms: cubic β-SiC and hexagonal α-SiC. In the RS fabrication process, the β-SiC phase is formed around the α-SiC particles^19^, acting as a bridging element that connects the discrete α-SiC particles, thereby creating a robust skeletal structure^20,21^, this significantly enhances the mechanical and thermal properties of the Al/SiC composites. SiC preforms can also be produced through conventional sintering (CS) in an ambient atmosphere^22,23^, during which SiO_2_ is produced on the surface of SiC particles due to oxidization^15,24^. While research has largely centered on the impact of the SiO_2_ layer on the mechanical properties of Al/SiC composites, the influence of silica on thermal properties has been less investigated^24^. Moreover, the silica precipitates can enhance the wettability between SiC particles and molten aluminium in pressureless infiltration, while simultaneously suppressing the formation of the detrimental Al_4_C_3_ phase^25,26^.

It is imperative to acknowledge that during the aluminium infiltration process, interfacial chemical reactions can promptly take place, leading to the formation of undesirable reaction products such as Al_4_C_3_ and Al_4_SiC_4_^27^. This occurs due to the thermodynamic instability of SiC at temperatures exceeding the melting point of aluminium^27–29^. As previous reports^9,27,30,31^, the threshold values for carbon activity necessary for the formation of Al_4_C_3_ are relatively low, owing to the limited solubility of carbon in molten aluminium. When introduced into the liquid alloy, carbon atoms react with aluminium, resulting in the formation of Al_4_C_3_, which can coat SiC particles either continuously or discontinuously. These adverse reactions lead to the substantial degradation of SiC particles and compromise the mechanical properties of the final product. Earlier studies^31^ have indicated that the formation of Al_4_C_3_ drastically reduces the material's TC to approximately 65 W/m K and its CTE to around 13.5 × 10^–6^/°C. Moreover, the Al_4_C_3_ phase has been found to be prone to moisture-induced corrosion. The gradual hydrolysis of Al_4_C_3_ in atmospheric moisture leads to the formation of Al-hydroxide, which ultimately accelerates crack propagation within the material.

Another factor for the Al_4_C_3_ generation is the relatively long infiltration period^31,32^. Vacuum pressure infiltration is a well-known method for infiltrating molten aluminium into porous preforms to obtain Al/SiC composites^32–34^. This method is chosen for its high production rate, low cost, capacity for net shaping, and ability to achieve a high volume fraction of reinforcement^35,36^. The process requires minimal equipment, is easy to operate, and has a short infiltration time. By manipulating processing parameters such as temperature, pressure, and duration, the formation of deleterious interfacial reactions can be obviated^35^. Moreover, the vacuum and high-pressure conditions under which this process is conducted help to mitigate shrinkage defects within the composite matrix, thereby enhancing its mechanical and thermal characteristics^32^. Zhu et al.^33^ explored the interfacial structure and stability of a co-continuous Al/SiC composite produced via vacuum pressure infiltration. Findings indicated that this technique promotes densification in the composites and averts the precipitation of Al_4_C_3_, leading to a continuous boundary devoid of precipitates or voids. Separate research validates that the TC of Al/SiC composites can be elevated to about 220 W/m k by incorporating bimodal size-distributed reinforcers through vacuum pressure infiltration^36^.

Previous studies have investigated the correlations between SiC particle size distribution, particle volume fraction, and thermal properties^36–40^. However, these studies were incomplete and unsystematic because they focused on only one aspect of the composite properties, such as TC, CTE, or bending strength. Molina and colleagues^36^ found that Al/SiC composites made from SiC mixtures with a mono-modal size distribution (average particle size is 170 μm) achieved the highest TC of 216 W/m k. Furthermore, the employment of bimodal SiC powders, with average particle sizes of 170 μm and 16 μm, facilitated the production of composites with elevated volume fractions, yielding Al/SiC composites with a TC as high as 220 W/m k^36^. Song and co-authors^15^ recently prepared Al/SiC composites with a high volume fraction of ceramic phase using SiC mixtures with a tri-modal size distribution (with an average particle size of 90 μm, 60 μm, and 10 μm). This study focused on elucidating the impact of pre-oxidation of SiC particles on the mechanical attributes of the composites, revealing that pre-oxidization can enhance the mechanical properties of the composites to a certain extent.

Currently, researchers have mainly focused on the pre-treatment of the SiC particles and the optimization of the aluminization process to improve the thermal conductivity of Al/SiC composites, but little attention has been paid to the post-treatment of the sintered SiC preforms. Addressing this gap, a comprehensive study was conducted here to explore the influence of the interfacial chemical reaction control, the volume fraction of preforms, and the specific post-oxidization of RS preforms on the thermal and physical properties of Al/SiC composites fabricated via reaction sintering together with vacuum pressure infiltration. The study compared the advantages of RS preforms with those of CS sintered preforms, delved into the effects of post-oxidization treatment and particle size distribution on TC and CTE were also investigated, and assessed the interfacial microstructure and physical properties of both the green and sintered samples.

## Results and discussion

The thermal behaviors frequently evaluated for Al/SiC composites are TC and CTE, which are tightly related to the interfacial characteristics between matrix and reinforcement, and to the content and particle size distribution of the reinforcement^41–43^. This research aimed to investigate the relationship between fabrication methods (including sintering approaches, post-oxidization treatment, and particle size distribution) and the thermal and physical properties of Al/SiC composites.

### Preparation of SiC porous preforms

**Figure 1:**
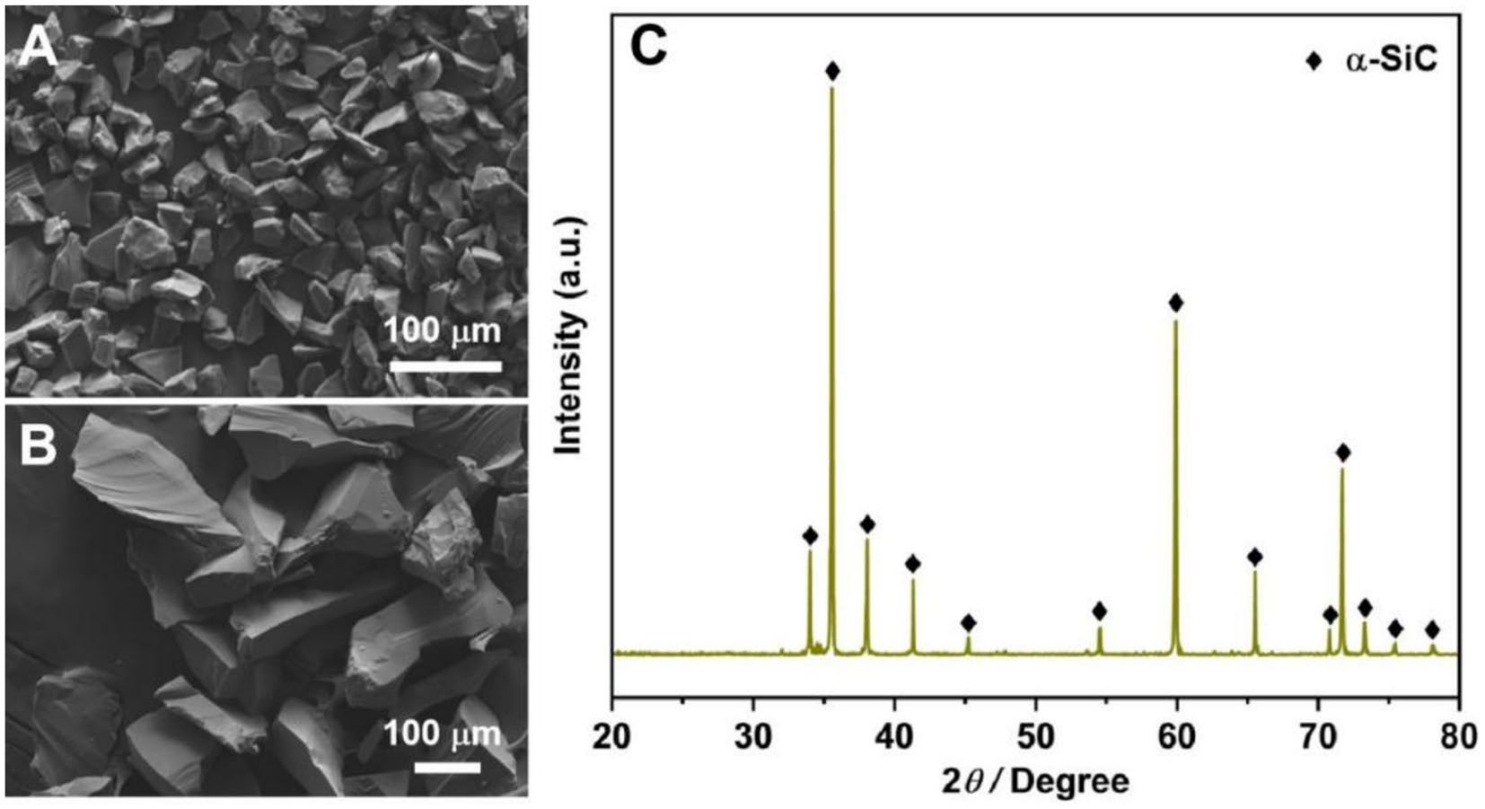
SEM images of raw SiC particles with different average particle sizes, (**A**) 14 μm, (**B**) 140 μm. (**C**) XRD pattern of 140 μm SiC particles.

**Figure 2:**
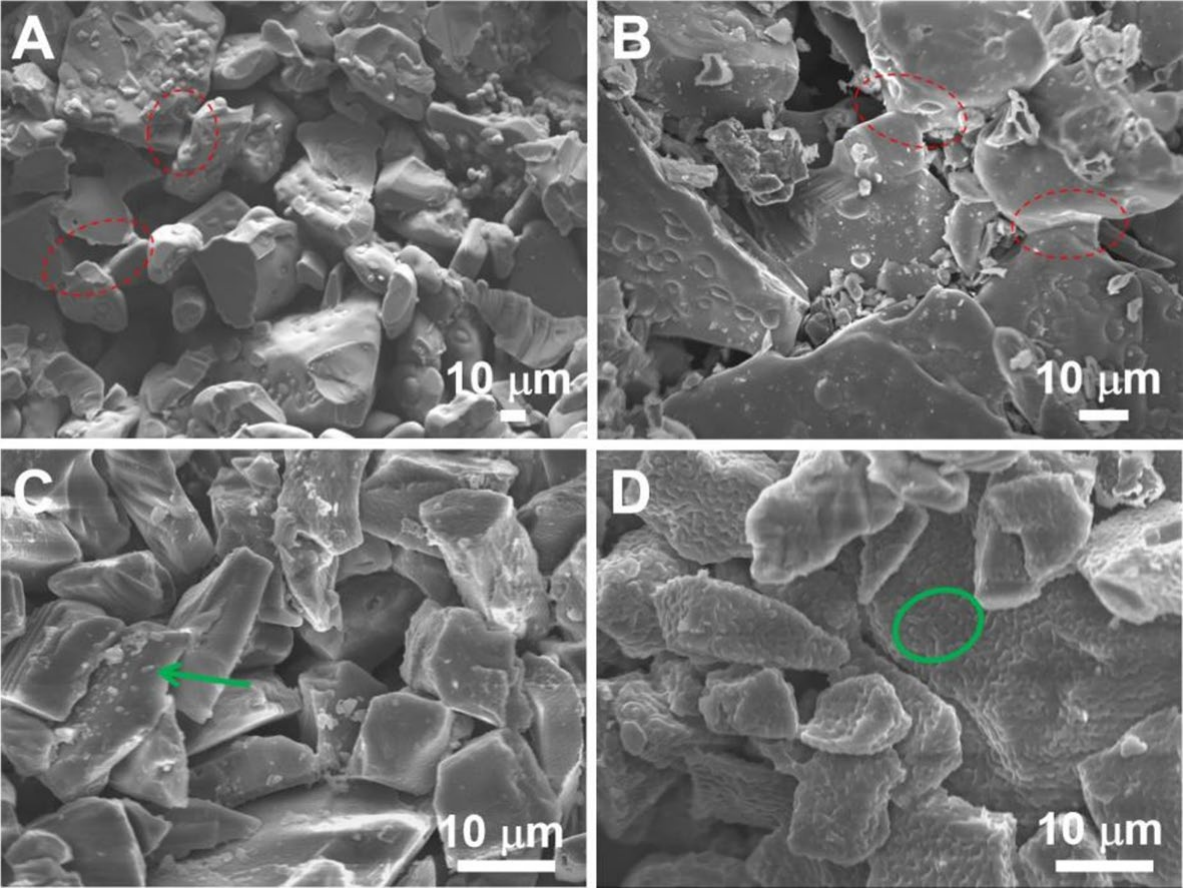
SEM images of fracture surface of SiC performs sintered at different situations, (**A**) RS in vacuum at 1550 °C, (**B**) RS in vacuum at 1700 °C, (**C**) CS in air at 1200 °C, (**D**) CS in air at 1300 °C.

Figure 1A and B show the SEM images of the raw SiC particles with an average size of 14 μm and 140 μm, respectively, employed in this study. Figure 1C depicts the XRD pattern of the 140 μm SiC particle, indicating that the predominant phase of it is α-SiC. The morphologies and phase compositions of SiC particles of varying sizes exhibit similar characteristics. Table 1 displays the bending strength (BS) and TC of samples sintered by CS in air and RS in vacuum at different sintering temperatures before and after aluminium infiltration. The BS of SiC preforms exhibits an upward trend with increasing sintering temperature, irrespective of whether the preforms were sintered through CS (CS preform) or RS (RS preform). In particular, the BS of the CS preforms sintered at 1100 °C, 1200 °C, and 1300 °C are 2.4 MPa, 4.8 MPa, and 10.3 MPa, respectively, while those for RS preforms sintered at 1550 °C and 1700 °C are 16.5 MPa and 21 MPa, respectively. The Al/SiC composites derived from CS preforms are designated CS composites, whereas those from RS preforms are referred to as RS composites. The BS values of CS composites sintered at 1100 °C, 1200 °C, and 1300 °C are 267.4 MPa, 296.1 MPa, and 310.2 MPa, respectively, with an increasing trend as the sintering temperature rises, yet remaining lower than those of RS composites. The RS composites sintered at 1550 °C and 1700 °C exhibit BS values of 325.6 MPa and 316.7 MPa, respectively. The possible reason for the CS specimens showing lower BS compared to RS specimens is the lower sintering temperature employed in the CS preform preparation and the relatively lower volume fraction of the preforms. Upon examination of the SEM fracture surface in Fig. 2, the 1700 °C RS sample exhibits more and wider sintering necks (marked with red circles) compared to the 1550 °C RS sample. Conversely, such features are notably absent in the CS samples. This suggests that higher sintering temperatures lead to stronger bonding between adjacent SiC particles, thereby creating robust interparticle bonding and higher bending strength of the preforms. Nevertheless, it was observed that the RS composites fabricated using preforms at 1700 °C and 1550 °C exhibited comparable TC and BS values, likely due to the equivalent density and volume fraction of the preforms (Table 1). In light of the sintering temperature, we employed a lower sintering temperature, 1550 °C, for our study.

**Table 1:** Physical and thermal properties of SiC preforms and Al/SiC composites sintered by CS and RS at different sintering temperatures.

Sintering temperature (°C)	CS			RS	
	1100	1200	1300	1550	1700
Volume fraction of preform (vol%)	60.1 ± 0.36	62.4 ± 0.33	63.3 ± 0.41	66.5 ± 0.39	66.7 ± 0.42
Density of preform (g/cm^3^)	1.92 ± 0.03	1.99 ± 0.03	2.03 ± 0.04	2.12 ± 0.06	2.14 ± 0.07
BS of SiC preform (MPa)	2.4 ± 0.5	4.8 ± 0.8	10.3 ± 2.1	16.5 ± 3.4	21.0 ± 4.1
BS of Al/SiC composite (MPa)	267.4 ± 10.5	296.1 ± 13.2	310.2 ± 12.0	325.6 ± 10.5	316.7 ± 13.4
TC of Al/SiC composite (W/m K)	152.5 ± 9.5	132.4 ± 6.7	111.4 ± 7.2	196.7 ± 8.3	194.3 ± 9.6

**Figure 3:**
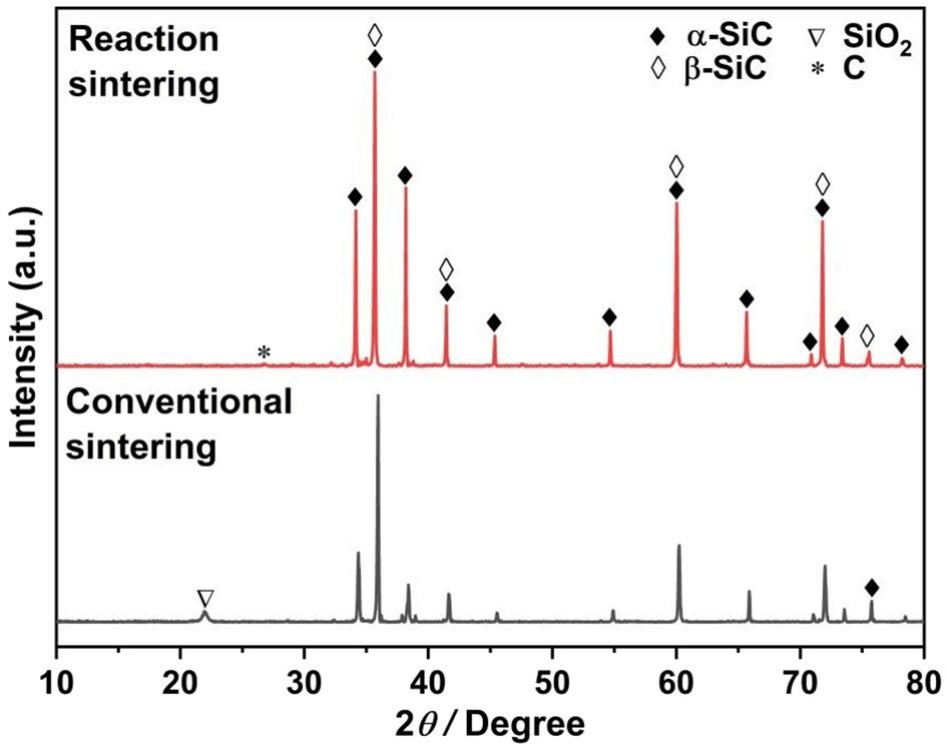
XRD patterns of RS preform (top) and CS preform (bottom).

**Figure 4:**
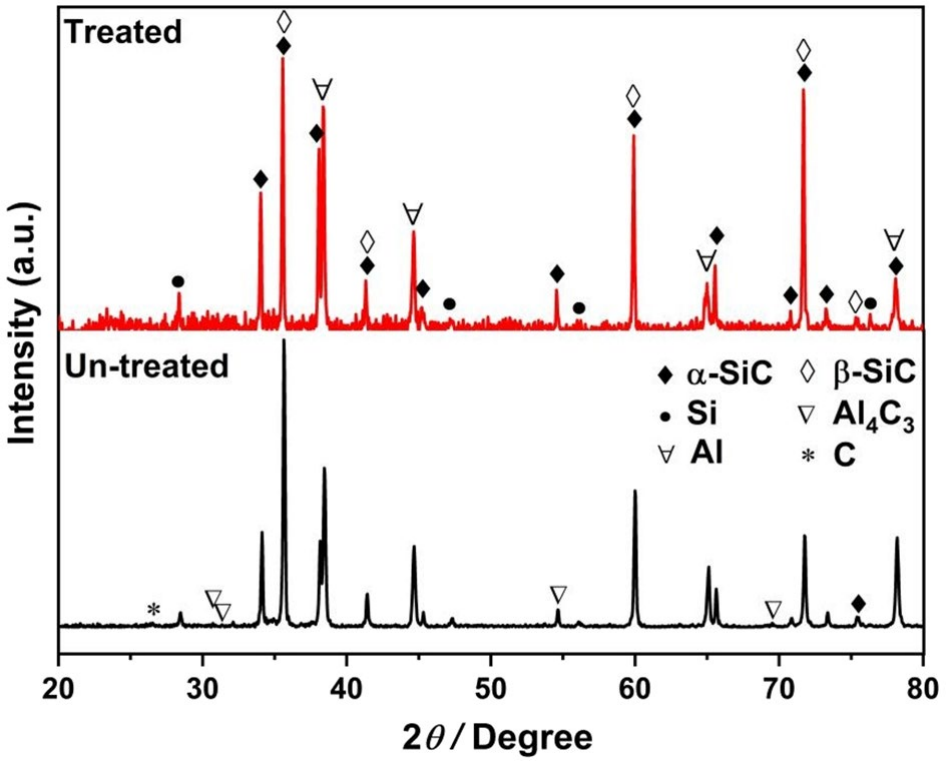
XRD patterns of Al/SiC composites, the sample treated by post-oxidization is shown at the top, the un-treated sample is shown at the bottom.

Again, Table 1 presents the TC data for Al/SiC composites. The TC values for CS composites sintered at 1100 °C, 1200 °C, and 1300 °C are 152.5 W/m K, 132.4 W/m K, and 111.4 W/m K, respectively, indicating a decrease in TC with ascending sintering temperatures during the preparation of CS preforms. A comparative SEM analysis of SiC fracture surfaces (Fig. 2C,D) indicates the formation of precipitates (denoted by green arrow and circle) on surfaces of samples sintered using CS at 1200 °C and 1300 °C. The XRD characterization, illustrated at the bottom of Fig. 3, identifies these precipitates as SiO_2_. To further confirm the presence of SiO_2_, EDS scans were performed on different samples, and the results are shown in Fig. S1. The sample sintered at 1300 °C exhibits a greater quantity of SiO_2_ precipitates as compared to the 1200 °C sintered sample. Additionally, the XRD result of the raw SiC particles shown in Fig. 1C confirms the predominance of the α-SiC, with no detectable SiO_2_ phase, suggesting that SiO_2_ is produced through the surface oxidization of SiC particles. The extent of SiO_2_ formation is found to be temperature- and condition-dependent, with higher sintering temperatures in air potentially enhancing oxidization and subsequent SiO_2_ formation. This is supported by the semi-quantitative analysis of the XRD results presented in Table 2 and Fig. 3 and Fig. S2, which reveal the SiO_2_ phase contents to be 2.9 wt%, 4.1 wt%, and 10.5 wt% for CS at 1100 °C, 1200 °C, and 1300 °C, respectively. Heat transfer in solid materials is predominantly mediated by phonon scattering, which is significantly influenced by the thermal interfacial resistance at material interfaces^44^. It is widely recognized that interface impurities, such as SiO_2_ and Al_4_C_3_, along with stress cracks and defects, have significantly detrimental impacts on TC behavior^12,44,45^. In the case of RS preforms, the introduction of Si powder and a carbon source into the initial mixture promotes the formation of the β-SiC phase, as evidenced by the XRD pattern observed in the top section of Fig. 4. As depicted in Figs. 2 and 3, the interfaces of RS preforms are free of silica inclusions due to their oxygen-free sintering conditions, containing only the SiC phase. Consequently, the TC values of RS composites significantly exceed those of CS composites, with values of 196.7 W/m K for the 1550 °C sintered sample and 194.3 W/m K for the 1700 °C sintered sample. Based on these findings, the sintering protocol for SiC porous preform fabrication has been refined, with subsequent experiments employing RS at 1550 °C to achieve pristine, robust, and highly thermally conductive products.

**Table 2:** The silica content of SiC preforms sintered by CS and RS at different sintering temperatures was determined by semi-quantitative analysis of XRD data (shown in Fig. S2).

	Before sintering	CS			RS	
Sintering temperature (°C)	/	1100	1200	1300	1550	1700
Silica content (wt%)	/	2.9 ± 0.4	4.1 ± 0.8	10.5 ± 2.8	/	/

The slash sign “/” represents that the phase content is below the detection limit of the XRD instrument.

### Post-oxidization for RS preforms

To implement the reaction sintering, a graphite crucible was used as the sample container. Green preforms were interposed with graphite papers before being loaded into the crucible. To fabricate the RS preform green bodies, Si powders (as the Si source) and PF resin (as the C source) both mixed with raw SiC powders to create the starting mixtures for die pressing. The RS process involved the chemical reaction between Si and C. In order to ensure an adequate reaction of silicon, it is necessary to introduce an excess of carbon sources. It is important to note that the RS was conducted in the graphite crucible, functioning as an additional carbon source. Therefore, a post-oxidization treatment in air is necessary to eliminate any remaining carbon and purge the particle surfaces. Otherwise, if residual carbon is present, it can react with molten aluminium during infiltration, forming the unstable and brittle compound Al_4_C_3_ (as shown at the bottom of Fig. 4). The instability and brittleness of the Al_4_C_3_ phase compromise the TC of Al/SiC composites, and its reaction with moisture degrades material properties^33^. To enhance thermal and mechanical attributes, preventing Al_4_C_3_ and employing post-oxidization treatment is essential.

**Figure 5:**
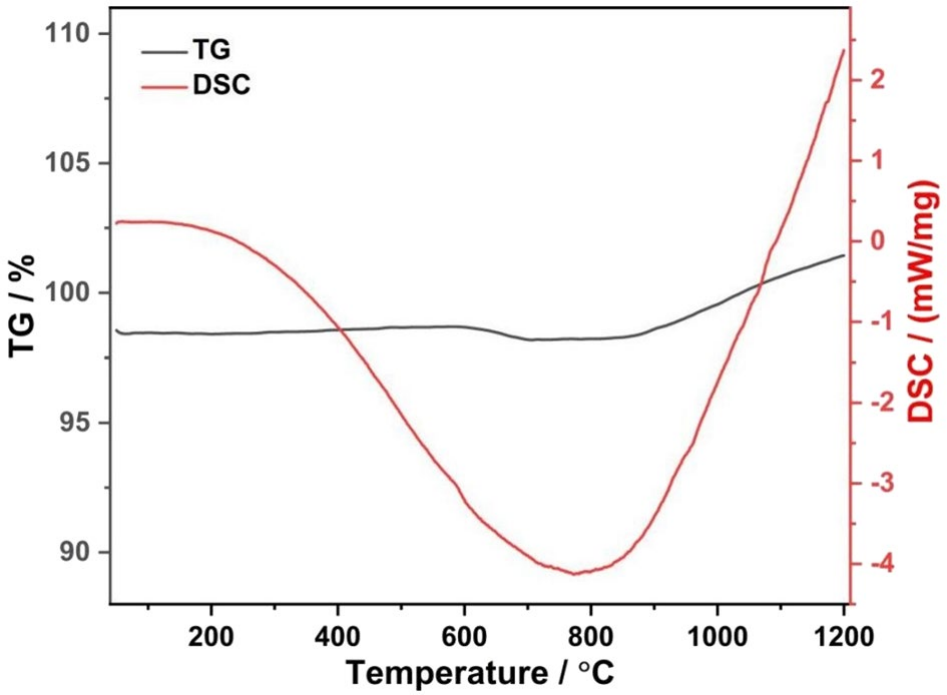
TG graph of RS preform before post-oxidization treatment.

**Figure 6:**
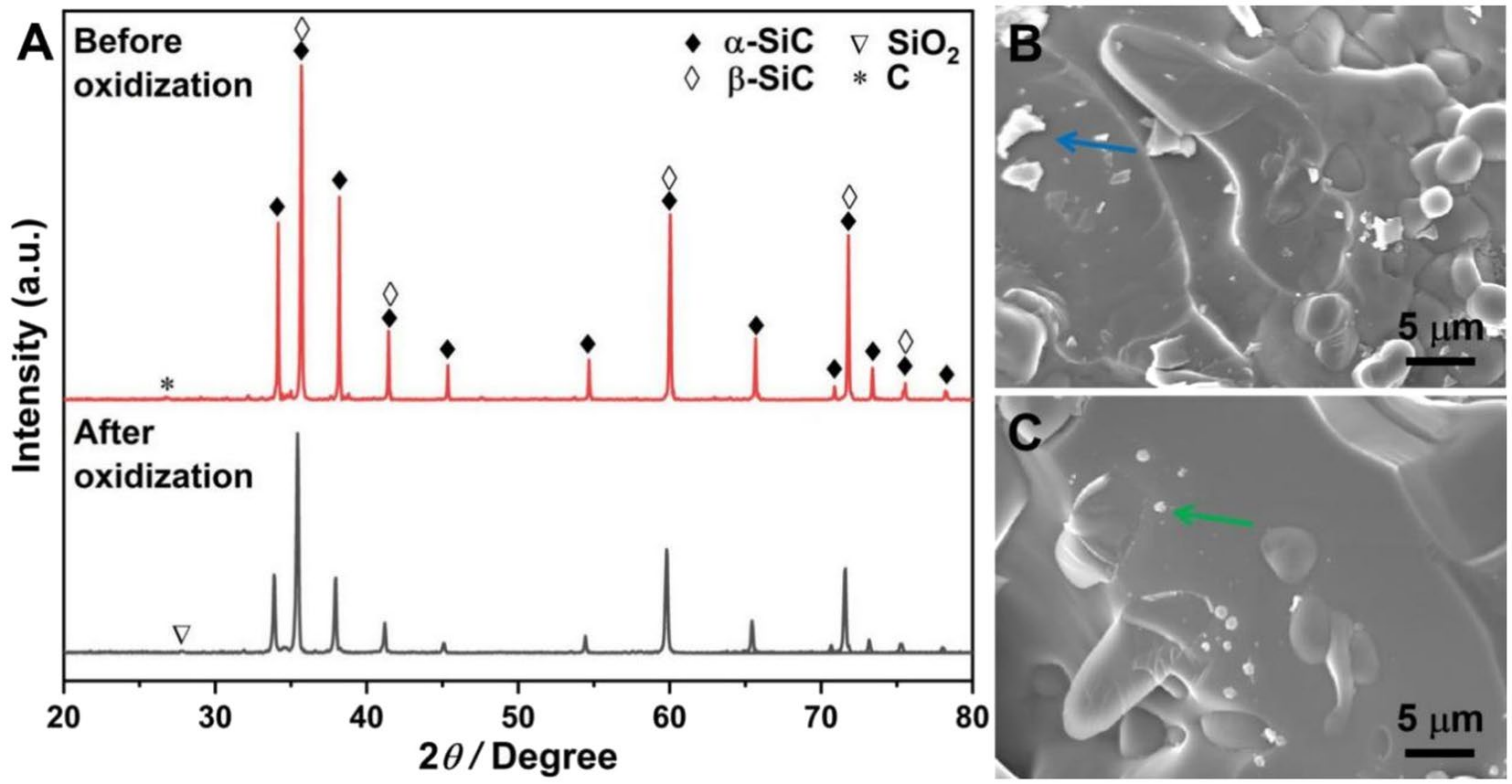
(**A**) XRD patterns of RS performs before (top) and after (bottom) post-oxidization at 800 °C, (**B**) SEM morphology of RS preform before post-oxidization, (**C**) SEM morphology of RS preform after post-oxidization at 800 °C.

The primary objective of the post-oxidization treatment for RS preform is decarbonization. This process, however, synchronously results in the formation of SiO_2_. Thermogravimetric (TG) analysis of the RS preform, devoid of post-oxidization treatment, as depicted in Fig. 5, reveals that the weight loss commences at roughly 575 °C and concludes at approximately 707 °C, signalling the carbon emission phase. Beyond 707 °C, an increase in mass is observed, indicating the generation of SiO_2_ on the SiC particle surface. Figure 6 displays SEM images and XRD patterns of the RS preforms before and after post-oxidization at 800 °C. As shown in Fig. 6A, the carbon signal vanished following the post-oxidization treatment, with the emergence of a SiO_2_ peak signifying the thorough elimination of carbon. For cross-verification of the residual carbon indicated by the blue arrow in Fig. 6B and the formed SiO_2_ highlighted by the green arrow in Fig. 6C, EDS spectral analyses were conducted on distinct specimens, and the data are provided in Figs. S3 and S4. Table 3 shows the semi-quantitative evaluation of newly formed SiO_2_ based on XRD data for RS preforms after post-oxidization at various temperatures for 2 h. Below 700 °C, the content of SiO_2_ falls below the instrumental detection threshold, indicating that carbon emission predominantly occurs within this temperature range. While temperatures exceeding 700 °C result in an inevitable content increment of SiO_2_, findings are in concordance with both TG and XRD analyses. With a mere 0.13 wt% increment in SiO_2_ content, the post-oxidization treatment temperature was optimally set at 700 °C, thereby guaranteeing the efficient elimination of residual carbon while minimizing the generation of additional SiO_2_. The presence of a minimal amount of SiO_2_ is helpful in enhancing the interfacial infiltration between the aluminium matrix and SiC particles^31^. Concurrently, the existence of SiO_2_ can impede the reaction between SiC and molten aluminium, effectively preventing the formation of Al_4_C_3_. Table 4 shows that the porosity of RS preforms increases from 26.8% before post-oxidization to 28.1% after post-oxidization. The heightened porosity facilitates the infiltration of liquid aluminium, contributing to a composite density enhancement of 2.4% (from 2.88 to 2.95 g/cm^3^) after post-oxidization (refer to Table 4). Notably, the BS and Young’s modulus (YM) remain invariant before and after post-oxidization. Inspection of the XRD patterns in Fig. 6A, which compares Al/SiC composites with and without post-oxidization treatment, reveals the absence of the Al_4_C_3_ phase in the treated sample, where the TC has escalated to roughly 209.4 W/m K. In stark contrast, the untreated sample exhibits the presence of both Al_4_C_3_ and carbon signatures, with a corresponding TC value of merely 196.7 W/m K. Evidently, the post-oxidization process has enhanced the composite’s TC by 6.5%. Overall, the post-oxidization technique effectively impedes detrimental interfacial reactions and promotes the thermal performance of the composite, accomplishing effective regulation of interfacial chemical reactions.

**Table 3:** The SiO_2_ content of RS preforms after post-oxidization at different temperatures for 2h.

Temperature (°C)	Preforms treated by post-oxidization at different temperatures			
	500	600	700	800
SiO_2_ content (wt%)	/	/	0.13 ± 0.02	0.57 ± 0.12

The content was determined by semi-quantitative analysis of XRD data. The slash sign “/” represents that the phase content is below to the detection limit of the XRD instrument.

**Table 4:** Physical and thermal properties of samples treated by post-oxidization at 700 °C or not.

	SiC preforms		Al/SiC composites			
	BS (MPa)	AP (%)	D (g/cm^3^)	BS (MPa)	YM (GPa)	TC (W/m K)
Un-treated	16.5 ± 3.4	26.8 ± 0.4	2.88 ± 0.08	325.6 ± 10.5	76.1 ± 4.7	196.7 ± 8.3
Treated	16.6 ± 3.3	28.1 ± 0.3	2.95 ± 0.03	323.4 ± 11.6	74.8 ± 3.6	209.4 ± 5.7

*AP* Apparent porosity, *D* Density.

### Particle size distribution and volume fraction of RS preforms

The thermal performance of Al/SiC is significantly influenced by the volume fraction, particle size, and particle size distribution within the SiC preforms, due to the finite metal/ceramic interface thermal resistance. To maximize the volume fraction of SiC compacts and thereby achieve superior TC and reduced CTE, it is imperative to optimize the particle size distribution. This optimization allows fine particles to envelop larger ones or fill voids, thereby enhancing packing efficiency^36,41^. Consequently, investigating the interplay between volume fraction and particle size distribution is crucial for fabricating Al/SiC composites with enhanced performance characteristics.

**Figure 7:**
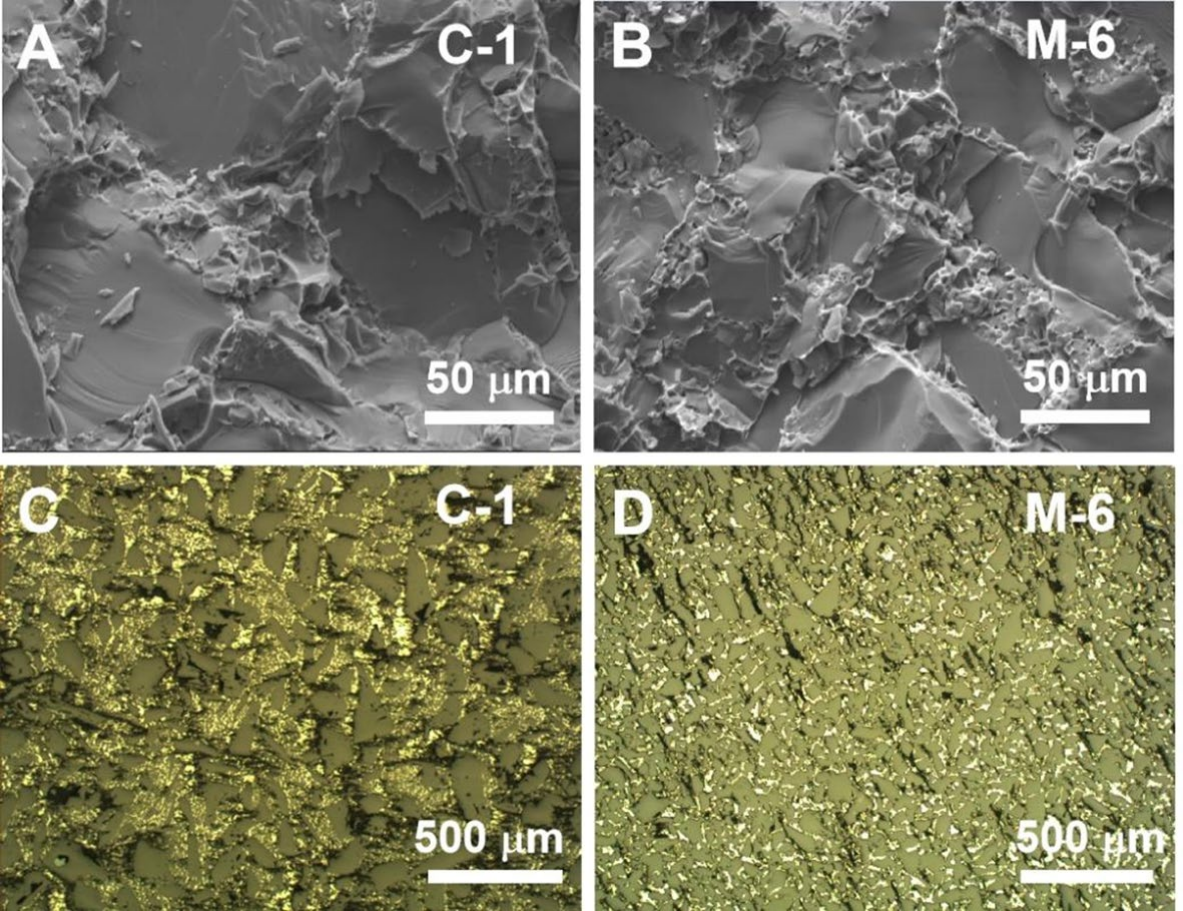
Transverse morphology images by SEM and optical microscope of Al/SiC composites: (**A**, **C**) for C-1, (**B**, **D**) for M-6.

In this study, SiC powders with diverse particle size distributions were employed to fabricate SiC compacts featuring a high volume fraction. Table 5 presents the compositions of the preforms labelled as C-1, C-2, C-3, C-4, C-5, and M-6, which were manufactured using mixtures of SiC powders with varying volume ratios of particles sized at 140 μm (coarse size), 90 μm (medium-1 size), 14 μm (medium-2 size), and 5 μm (fine size). This table also exhibits the volume fractions of the compacts after curing and after sintering. The highest volume fraction in C-1 preform indicates that the fine-sized particles do not positively contribute to the final volume fraction. The volume fraction of the M-6 preform is 7.6 percentage points lower than that of the C-1 preform, indicating that the incorporation of coarse particles contributes to an increased volume fraction, a finding proved by the fracture observations presented in Fig. 7. The SEM and optical observations of the C-1 specimen are shown in Fig. 7A and C, respectively, while Fig. 7B and D show the corresponding images for the M-6 composite. Analysis of the SEM images in Fig. 7A and B reveals that the primary fracture mode is transgranular fracture within the SiC particulates, satisfying the robust bonding between the particles and matrix. Metallographic images in Fig. 7C and D, depicting polished Al/SiC composites, show SiC particles in grey contrasted against the bright Al phase. The SiC reinforcements within the matrix are uniformly distributed, and no clustering is observed. Additionally, the Al phase is fully infiltrated, resulting in a dense and complete composite.

**Table 5:** Compositions of the starting blends and the volume fractions of the corresponding preforms.

	Mass fraction of compositions in blends (wt%)			Volume ratio of different size particles in blends (vol%)				Volume fractions of preforms (vol%)	
	SiC	Si	PF resin	Coarse (140 μm)	Medium-1 (90 μm)	Medium-2 (14 μm)	Fine (5 μm)	After curing	After sintering
C-1	92.9	9.1	The PF resin constitutes 7.5wt% of the combined mass of SiC and Si	2	–	1	–	74.63 ± 0.37	72.60 ± 0.24
C-2				2	–	1	0.5	71.92 ± 0.56	69.98 ± 0.49
C-3				3.5	–	1	0.5	73.32 ± 0.76	71.38 ± 0.47
C-4				4.5	–	1	0.5	73.56 ± 0.71	71.64 ± 0.48
C-5				5.5	–	1	0.5	70.98 ± 0.50	68.97 ± 0.63
M-6				–	2	1	–	68.03 ± 0.47	66.50 ± 0.39

**Figure 8:**
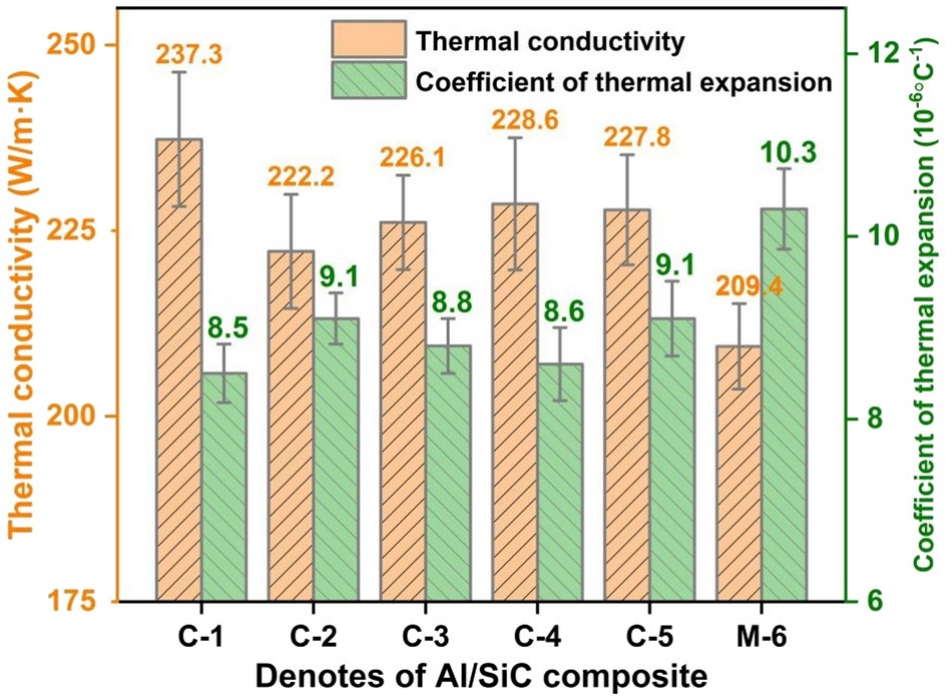
The effect of volume fraction and particle size distribution on TC and CTE of Al/SiC composites.

**Figure 9:**
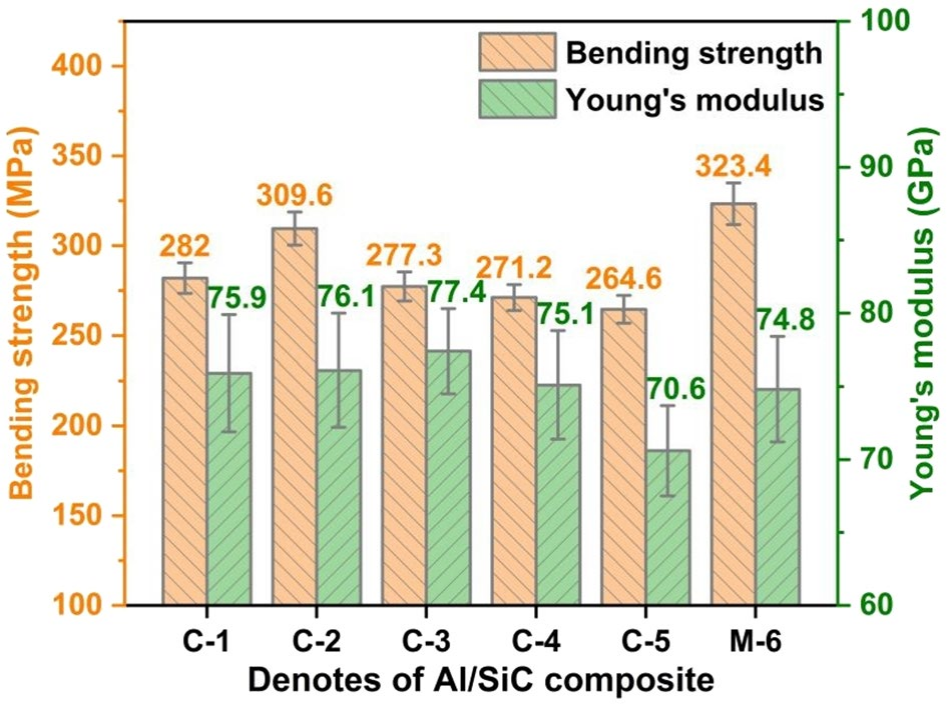
The effect of volume fraction and particle size distribution on BS and YM of Al/SiC composites.

Figure 8 displays the TC and CTE of Al/SiC composites with varying SiC particle size distributions. Notably, the C-1 composite exhibits a superior TC of 237.3 W/m K, which is significantly higher than that of the M-6 sample (209.4 W/m K) and other variants. This enhancement can be primarily attributed to two factors: Firstly, the increased particle size from 90 to 140 μm results in a rising in the volume fraction of sintered SiC preform from 65 to 72.6 vol%. It is established that smaller particles introduce more interfaces within the same volume fraction compared to larger particles. Consequently, the second factor is likely that larger particles reduce both the quantity and contact area of interfaces among adjacent particles, diminishing interface thermal resistance and thus enhancing TC values. Typically, aluminium exhibits a higher CTE than SiC, and for Al/SiC composites, the requirement is to achieve a high TC coupled with a low CTE. A higher volume fraction corresponds to a greater proportion of the SiC phase, which has been shown to have a beneficial impact on TC, and a contrasting effect on CTE. As observed in Fig. 8, the M-6 composite displays the highest CTE behaviour among the specimens. Therefore, to optimize thermal properties, it is advisable to minimize or avoid the inclusion of fine size particles in Al/SiC composites. Conversely, larger particles contribute to an increased SiC preform volume fraction, which enhances TC and reduces CTE of the composite. Figure 9 displays the BS and YM of Al/SiC samples fabricated using SiC powders with varying size distributions. Figure S5 shows the typical stress–strain curves of C-1 and M-6 samples. The findings suggest that a higher volume fraction and larger particle sizes are not conducive to superior mechanical properties. Among the samples tested, M-6 exhibited the highest BS (323.4 MPa) and YM (74.8 GPa). This can be ascribed to that the SiC phase in Al/SiC composite acts as a brittle phase, and a high content of such a phase does not enhance mechanical performance^46^. Moreover, large particles fail to induce synergistic deformation with the matrix, leading to stress concentration and crack formation^46^. In contrast, smaller particles within the matrix tend to the extend crack propagation path and absorb energy, thereby resulting in increased BS and YM. Collectively, the C-1 composite offers a promising compromise, achieving high thermal performance without substantial compromise to mechanical attributes in this study.

## Conclusion

In summary, the Al/SiC composites with enhanced thermal conductivity and reduced coefficient of thermal expansion were successfully prepared using post-oxidization treatment based on reaction sintering coupled with vacuum pressure infiltration technique. Reaction sintering, as opposed to conventional sintering methods, effectively prevents the formation of substantial interfacial deposits. Importantly, the proposed post-oxidization treatment of RS preforms effectively regulated the interfacial reactions during aluminium infiltration. Subsequent to post-oxidization, the remaining carbon was entirely eliminated, resulting in a clean and neat RS preform and preventing the formation of Al_4_C_3_ in the next infiltration step. Furthermore, the thermal conductivity of the composite was further improved by increasing the volume fraction of SiC preforms through particle size grading. Our study reveals that the resultant Al/SiC composites possess a pristine interface and exhibit superior thermal conductivity and a lower thermal expansion coefficient. These findings offer valuable insights for manufacturing Al/SiC composites with superior thermal performance.

## Experimental procedure

### Materials

In this study, the aluminium alloy was commercially obtained ZL101A, the composition of which is detailed in Table 6. The commercial α-SiC powders with mean particle sizes of 5 μm, 14 μm, 90 μm and 140 μm were purchased from Jiangsu Leyuan Material Co., Ltd (China). The SEM and XRD results of the SiC particles are shown in Fig. 1, revealing a uniform particle distribution and confirming the predominant α-SiC phase. Table 7 presents the data on the oxidized impurities and carbon content in the raw SiC powder, as determined via UV–Vis spectrometer (UV-1800PC, Shanghai Mapada, China) and Infrared carbon/sulfur analyzer (EMIA Pro, Horiba, Japan), respectively, in accordance with GB/T 3045-2003 (Chemical analysis of silicon carbide).

**Table 6:** Chemical ingredients of ZL101A alloy.

Component	Si	Fe	Cu	Mn	Mg	Zn	Al
Wt%	6.5 ~ 7.5	0.2	0.1	0.1	0.25 ~ 0.45	0.1	Remaining

**Table 7:** Composition analysis of raw SiC particles according to GB/T3045-2003 (chemical analysis of silicon carbide).

Item	Percentage (wt%)
MgO	0.007
Al_2_O_3_	0.002
CaO	0.012
C	29.994
SiO_2_	0.119
SiC	99.21

### Composite preparation

**Figure 10:**
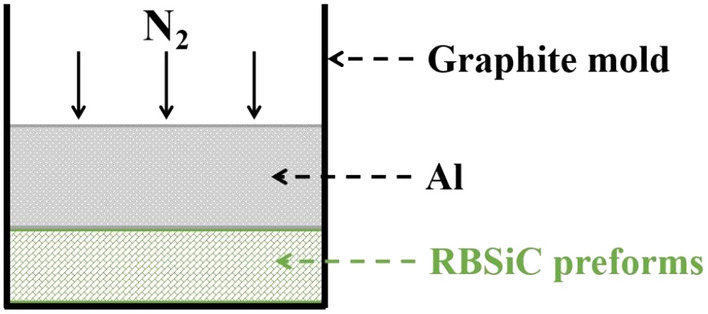
Schematic diagram of the infiltration mold for the vacuum pressure infiltration method.

For the preparation of composites, phenol–formaldehyde resin (supplied by Zhengzhou Hengtong Chemical Industry Co., Ltd, China) was selected to serve as the carbon source for reaction sintering (RS) and as a binder for the die pressing process. The SiC and Si powders, phenol–formaldehyde (PF) resin and benzenesulfonyl chloride (curing agent for PF) were thoroughly mixed by a planetary ball mill (Changsha Tianchuang Powder Technology Co., Ltd, China) equipped with SiC balls, operated at a rotational speed of 240 rpm for a duration of 4h, to produce the initial mixture for die pressing. The composition of the blends and the volume fraction of the preforms are shown in Table 5. The typical XRD pattern for the preform green body is shown in Fig. S6. The green SiC bodies fabricated by die pressing (180 kg/cm^2^, 40 s) was subsequently cured at 200 °C for 4h prior to being sintered at 1550 °C for 5 h in vacuum conditions at a programmed heating rate of 2 °C/min to obtain the RS preforms. To facilitate the RS process, the green SiC bodies were separated with graphite papers and then placed in a graphite crucible. After sintering, the RS preforms were cleaned in water by ultrasonication for 20 min. Then the RS preforms were treated by post-oxidization, that is, the preforms were treated in air at 700 °C for 2.5 h by using a tunneling furnace. Thereafter, molten aluminium was infiltrated into the RS porous preforms using the vacuum pressure infiltration method as illustrated in Fig. 10. This process was conducted by a ZYQ250/400-2.1000 furnace obtained from Aviation Industry in Western Sichuan Machinery Factory with an infiltration pressure of 5 MPa, and a duration time of 10 min.

For the SiC preforms sintered by CS in air, a starting mixture containing SiC powders and polyvinyl alcohol was prepared by ball milling as described above. Then, the SiC preforms were sintered in air in a muffle oven for 3 h at a tailored temperature. The selected sintering regimes were 1100 °C, 1200 °C and 1300 °C. The aluminium infiltration was similar to the above.

### Microstructure and mechanism characterization

Microstructure characterization of the composites was performed by scanning electron microscopy (SEM, JEOL JSM-5610LV, Japan) equipped with an energy dispersive spectrometer (EDS, Noran System Six, USA) to investigate the morphology and components of the transverse section of the preforms. The interfacial reaction products were identified by X-Ray diffraction (XRD) analysis using a SmartLab diffractometer (Rigaku, Japan). The corresponding ICDD card numbers for α-SiC, β-SiC, SiO_2_, Si, C, Al_4_C_3_, Al were referenced as 01-075-8314, 04-008-7773, 04-008-7812, 00-026-1481, 01-086-8296, 04-001-3358, and 04-012-7848, respectively. The semi-quantitative analysis of the XRD results was conducted using the Rietveld method via PDXL2 software, and each sample was measured three times to obtain the average value. The volume ratio, density and porosity of the prepared specimens were measured by the Archimedes’ method. The dimensions of the specimen prepared for the bending test were precision-cut to 22 × 4 × 2 mm. The bending strength and Young’s modulus were determined through a standardized three-point bending test conducted on a universal testing machine (model WD-P4504), adhering to ASTM E1890 guidelines. The test utilized a span length of 16 mm and maintained a controlled loading rate of 0.5 mm/min^47^.

### Thermal property characterization

Following ASTM guidelines, disk-shaped samples of 12.7 mm in diameter and 2 mm in thickness were sputter-coated with a black carbon coating. Detailedly, the TC (*κ* = *αρCp*) was calculated from individual measurements^12^ of the thermal diffusivity (α) using the laser flash method (Netzsch LFA 450, Germany) according to ASTM E1461 and the specific heat (*Cp*) by differential scanning calorimetry (DSC) with a Netzsch Pegasus 404 system (Netzsch, Germany) according to ASTM E1269, and the density (*ρ*) of prepared specimens was measured by Archimedes’ method.

The method of CTE measurement can be found elsewhere^21^, briefly involving plate samples sized 5 × 5 × 1 mm, as per ASTM E831. A thermomechanical analyzer in conjunction with a system purchased from TA Instruments (TMA450, USA) was used to determine the CTE in the range of 25 °C and 100 °C in air with a heating rate of 3 °C/min.

### Supplementary Information

Supplementary Figures.

## Data Availability

The datasets used and/or analyzed during the current study available from the corresponding author on reasonable request.
