# Supplementary material for: Improving thermal conductivity of Al/SiC composites by post-oxidization of reaction-bonded silicon carbide preforms
Source: Sci Rep. 2024 Jul 18;14:16610. doi: 10.1038/s41598-024-67653-y (PMC11258292; doi:10.1038/s41598-024-67653-y)
Supplement: Supplementary file 1 — Supplementary Figures. [file 41598_2024_67653_MOESM1_ESM.docx]

Supporting information for

**Improving thermal conductivity of Al/SiC composites by post-oxidization of reaction-bonded silicon carbide preforms**

Xinping Lin^1^, Qiang Xu^2^, Tianyou Deng^1^, Bingquan Yang^1＊^, Liang Chen^1＊^

Upon examination of Figures S1A and S1B, it is evident that the oxygen content of the SiC samples increases substantially from 2.46wt% (raw SiC particles) to 27.59 wt% (upon sintering at 1300°C) by conventional sintering process. This significant rise serves as compelling evidence for the oxidization of SiC. Figures S1C and S1D further illustrate that when SiC preforms are subjected to sintering at 1300°C, extensive and continuous deposits form on the particle surfaces. These deposit regions exhibit a marked oxygen content of 29.14 wt%, in stark contrast to the surrounding regions, which do not cover deposits containing only 7.33 wt% oxygen. Moreover, a comparative analysis of Figures S1E and S1F reveals that after sintering at 1200°C, SiC particles also display surface deposits with an oxygen content of 22.54 wt%, surpassing the oxygen content in the adjacent areas without deposits, which is 7.48 wt%. The above discussion suggests that the deposits are primarily composed of SiO_2_ oxidizing by SiC on the interfaces.


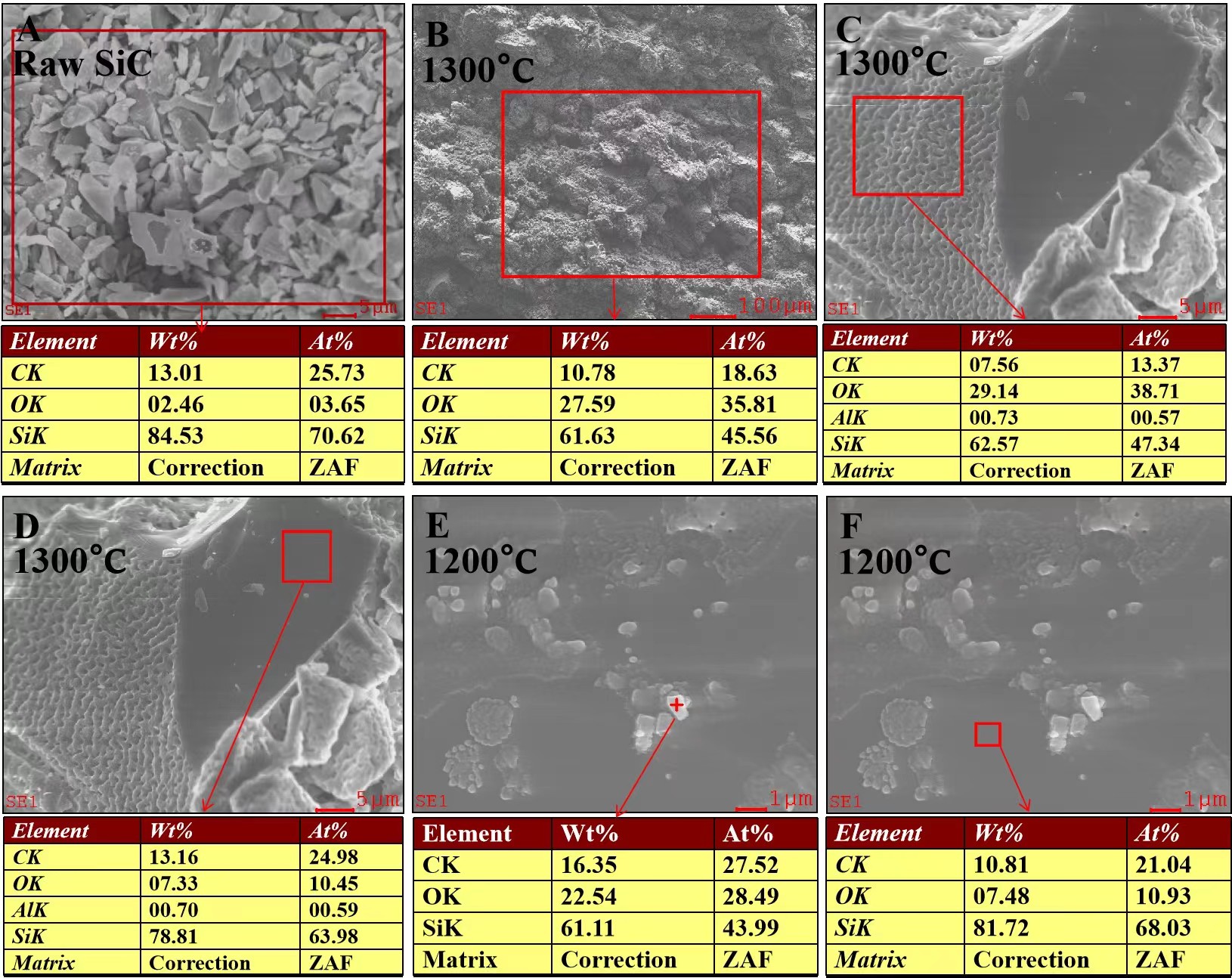


**Figure S1.** (A) EDS scan for raw SiC particles. EDS scans for CS preforms: (B) and (C) and (D) sintered at 1300°C, (E) and (F) sintered at 1200°C.


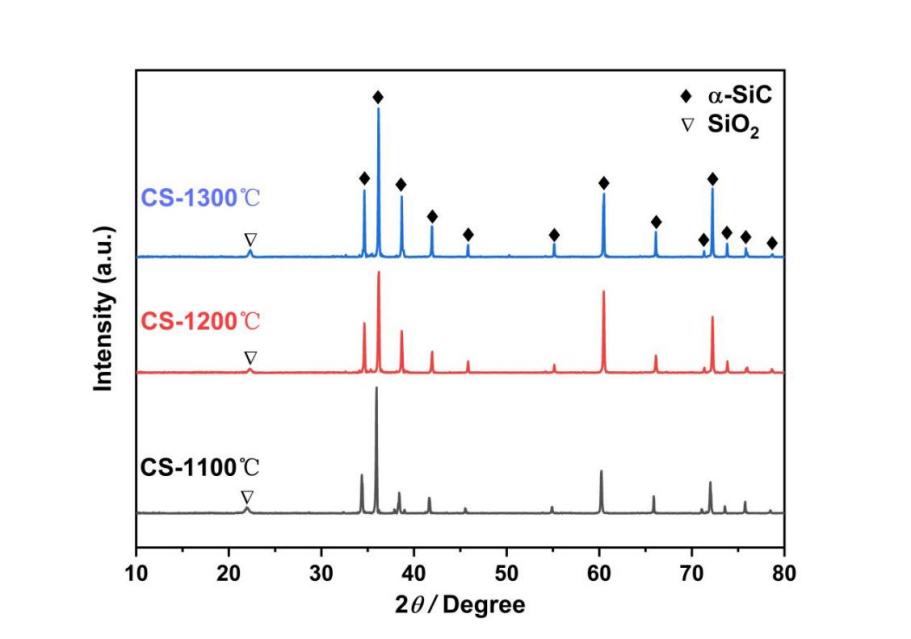


**Figure S2.** XRD patterns of CS preforms sintered at 1100°C, 1200°C, and 1300°C, respectively.

In Figure S3A, the oxygen content of the RS preform is initially measured at 2.97wt% prior to post-oxidization. Subsequent to the thermal treatment at 800°C, the oxygen content increases significantly to 8.78wt%, concomitant with a reduction in carbon content from 15.09wt% to 10.19wt%. Moreover, as depicted in Figure S4A, the prominent contaminant indicated by the blue arrow exhibits a carbon content of 55.99wt%. Following the post-oxidization process, the larger contaminants were effectively eliminated, giving rise to the formation of minute deposits (denoted by green arrow). These newly formed deposits exhibited an enhanced oxygen content of 8.32wt%, in contrast to the adjacent regions devoid of deposits. These observations indicate a transition characterized by the removal of residual carbon and the subsequent production of lightweight SiO_2_, attributable to surface oxidization.


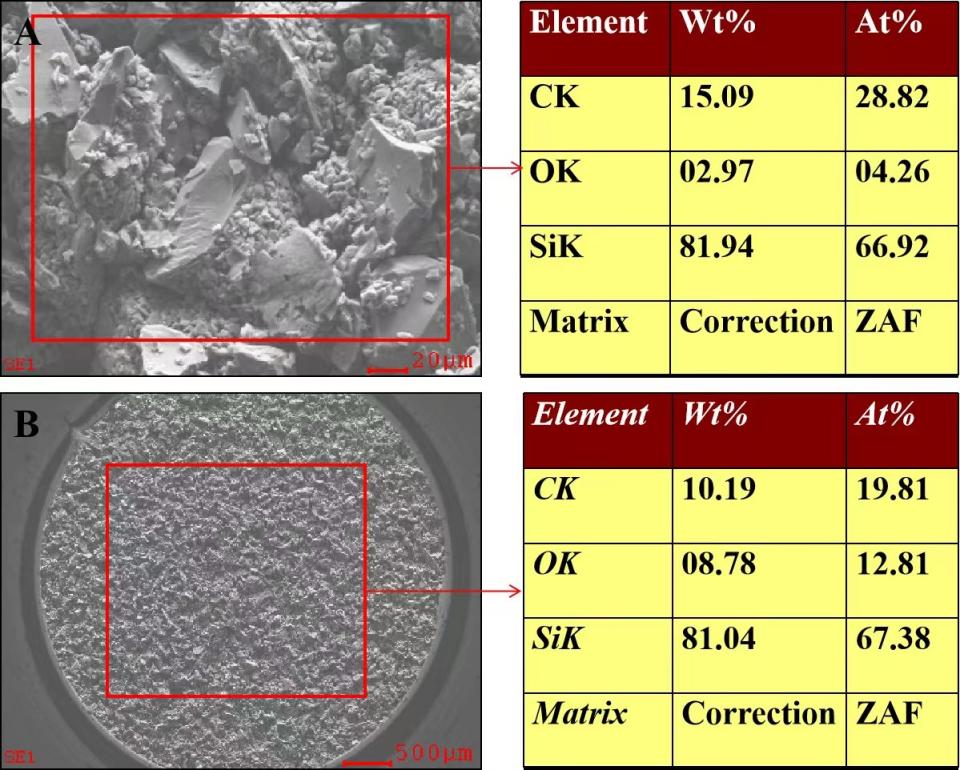


**Figure S3.** EDS spectral analyses of RS performs, (A) before post-oxidization, (B) after post-oxidization at 800°C.


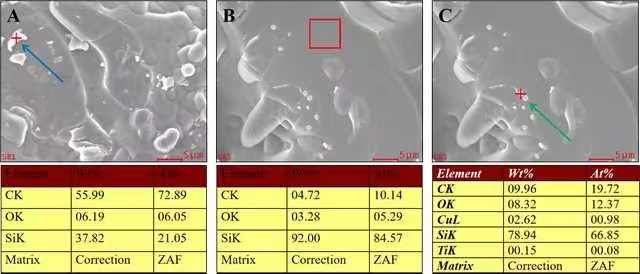


**Figure S4.** EDS spectral analyses of RS performs, (A) before post-oxidization, (B) and (C) after post-oxidization at 800°C.


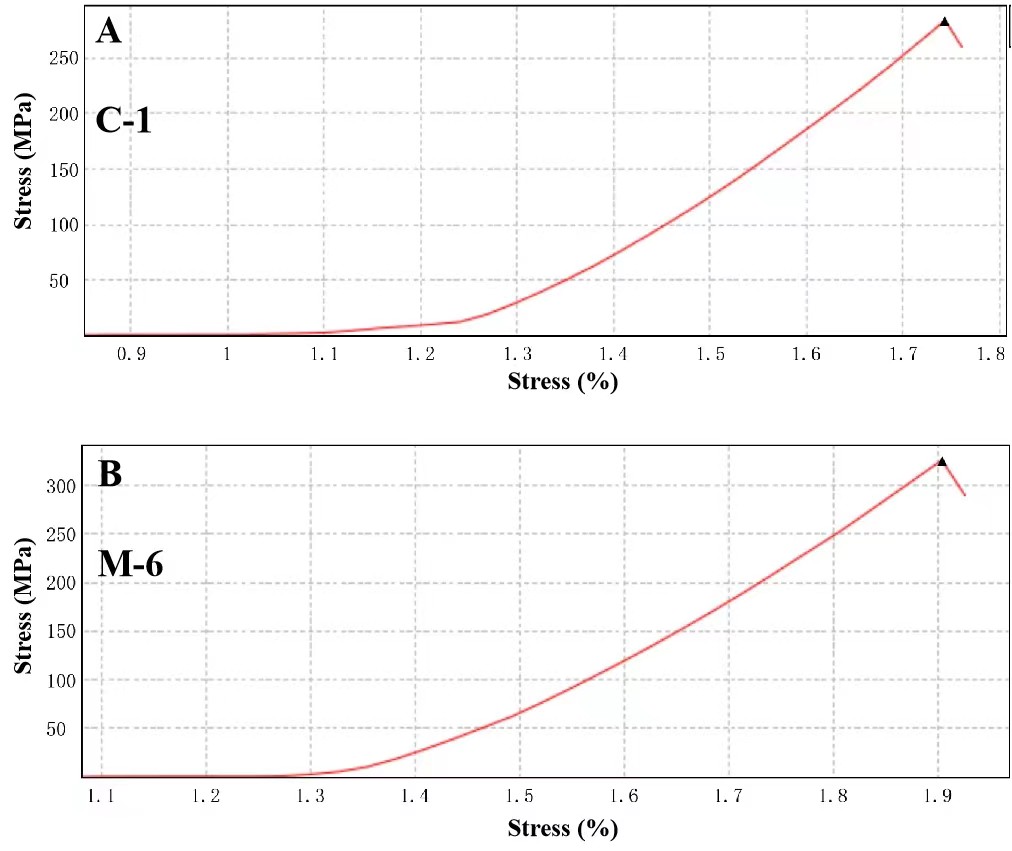


**Figure S5.** The typical stress-strain curves of (A) C-1 and (B) M-6 samples.


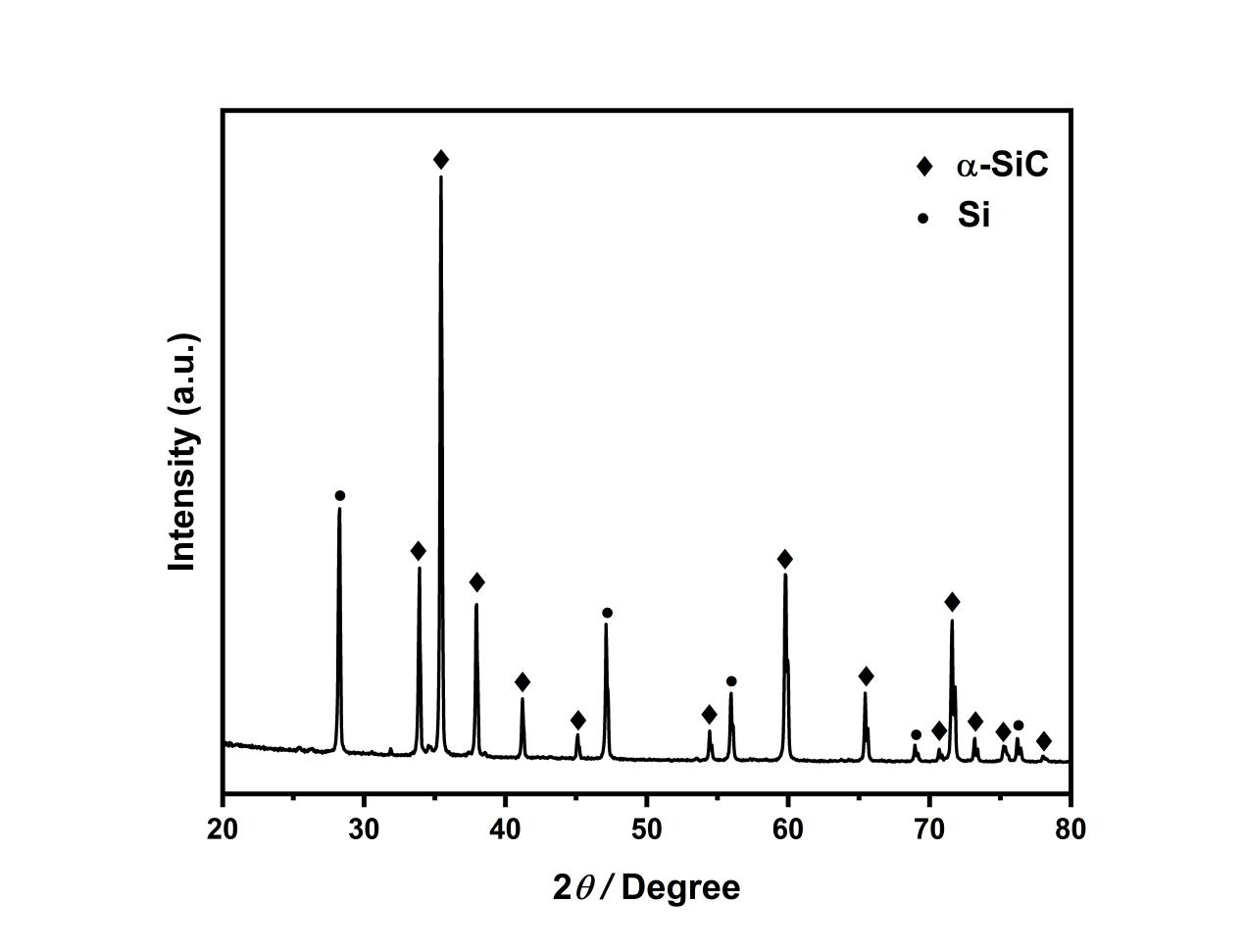


**Figure S6.** The XRD pattern for the preform green body.
